# Development of the Faith Community Child Protection Scale with Faith Leaders and their Spouses in Senegal, Uganda and Guatemala

**DOI:** 10.1007/s10943-022-01660-z

**Published:** 2022-10-15

**Authors:** K. Diaconu, K. Jailobaeva, T. Jailobaev, C. Eyber, A. Ager

**Affiliations:** Institute for Global Health and Development, Queen Margaret University, Edinburgh, EH21 6UU UK

**Keywords:** Child protection, Psychometric measure, Faith communities, Child rights, Physical punishment, Factor analysis

## Abstract

Violence, abuse and neglect constitute major threats to children’s health and wellbeing globally. However, until recently, relatively little systematic attention has been paid to the role of faith communities in shaping the protective environment for children. This paper describes the development of a measure to capture child-protective disposition amongst faith communities through field studies with faith leaders and their spouses in Senegal, Uganda and Guatemala. Identifying common factors related to child care and protection practices, orientation to child rights and approaches to discipline, the measure potentially serves to both inform and evaluate interventions seeking to engage with the beliefs and behaviours of faith communities to support children’s health and wellbeing.

**Supplementary Information:**

The online version contains supplementary material available at 10.1007/s10943-022-01660-z.

## Introduction

Addressing violence against children is of paramount importance. In relation to children aged 2–4 years, the United Nations Children’s Fund (UNICEF) estimates that 75% of children ‘are regularly subjected to violent discipline (physical punishment and/or psychological aggression) by their parents or other caregivers’; over 60% are further ‘subjected to physical punishment’ (UNICEF, 2017, p. 7). At least one billion children experience violence annually (End Violence against Children, 2020).

In 2011, General Comment 13 of the Committee on the Rights of the Child elaborated the implications of Article 19 of the Convention of the Rights of the Child (CRC) regarding the right of the child to freedom from all forms of violence (UN & CRC, 2011). The subsequent decade has been marked by increased advocacy in this area. Ending violence against children is a major focus of UNICEF strategy (UNICEF, 2017), and the Global Partnership to End Violence against Children has mobilised over $68 m since its establishment to invest in programming (End Violence against Children, n.d.).

Various analyses document the embedded nature of violence, abuse and neglect within societies (UNICEF, 2014). Use of a social ecological lens draws attention to the role of caregivers, families, schools and other local mechanisms in establishing a more protective environment (Ager et al., 2010). However, until recently, little systematic attention has been paid to faith communities that, in many societies, have a major impact on attitudes and behaviour toward children (Hanmer & Robinson, 2012; Marshall & Mui, 2016; Robinson & Hanmer, 2014).

The Kyoto Declaration, adopted by almost 1,000 religious leaders from all world religions in Japan in 2006, outlines the ways religious communities can work to eliminate violence against children. Key actions include inter-religious cooperation, the use of religious texts to teach about child rights, advocacy and awareness against violence, and educating and supporting families and communities to care for children holistically (Dodd & Robinson, 2010). The resources and values of faith communities potentially support many elements of community-based child protection response, including: a volunteer base, wide networks and capacity to mobilise resources (Ager et al., 2015). The role of faith communities in shaping social norms is increasingly acknowledged (Levin, 2020). Effective engagement with faith community resources is especially crucial in low- and middle-income settings where central and local governmental capacities for formal children protection services are typically severely limited (Ward et al., 2016). Given their potential influence, this has encouraged programming approaches in such settings that explicitly target faith leaders as a conduit to broader community change processes (Eyber et al., 2018).

Efforts to better understand and, where appropriate, shape the influence of faith leaders require appropriate measures of the views held within faith communities regarding children and childhood. In advance of intervention the first step needs to be an appraisal of the current outlook of the faith community with regard to childhood. This includes its understanding—informed by religious belief and practice—of child protection, violence against children, child rights and the roles and responsibilities of parents, religious leaders and the wider community. Partnership with local faith communities and design of context-sensitive interventions critically depends on this assessment to inform the direction of programming and identification of locally accepted actions to strengthen child protection (Palm & Eyber, 2019). Qualitative, ethnographically informed approaches are clearly important here. However, quantitative measures potentially provide a basis for tracking change over time and, in some circumstances, relevant comparison across settings.

This paper reports on the development of a survey tool—the Faith Community Child Protection Scale—through field studies with faith leaders and their spouses in selected districts of Senegal, Uganda and Guatemala. We outline the development and refinement of the scale through psychometric analysis and provide recommendations for its use and adaptation. The aim is to provide a tool for use by child protection practitioners and researchers globally that will inform faith-sensitive programming in this field (Ager et al., 2019) and help strengthen the surrounding evidence base.

## Methods

### Settings

Field studies were completed in Senegal in March 2016, in Uganda between April and May in 2018, and in Guatemala in September 2019. In Senegal three communities in Missirah District were surveyed and one community in Katakel District. Populations in these locations were largely Muslim, broadly mirroring the religious background of the nation (90% Muslim, 5% Christian and 5% others; Gifford, 2016). Preliminary community assessments supported the view that faith leaders in Senegal significantly influence beliefs, values, attitudes and behaviours in communities, including with regard to marriage practices, gender roles and the role of families, children and youth (Jailobaeva et al., 2021).

The communities surveyed in Uganda were from Buikwe, Kalongo, Kyalulangira, and Mpigi Districts. The level of religious affiliation in Uganda is high, with the overwhelming majority of the population (over 99%) declaring a religious affiliation (Uganda Bureau of Statistics, 2016). According to the most recent census data (Uganda Bureau of Statistics, 2016), 85% of people are practicing Christians (Catholics 39%, Anglicans 32%, and Pentecostals/Evangelicals 11%) and 14% are Muslims. The religious profile in the districts surveyed reflected the national picture. Initial community assessments indicated that faith leaders participated in the promotion of child wellbeing activities in their communities through influencing beliefs and behaviours of community members (Jailobaeva et al., 2021).

The Guatemalan communities surveyed were drawn from Jocotán, San Juan Ermita and Camotán Districts. Guatemala is predominantly a Christian country, with 90% of people self-identifying as Christian. Historically, the Catholic faith dominated the religious landscape. However, since the 1970s Protestantism has been expanding rapidly and now constitutes over 40% of the population (Bjune, 2016). The Mayan religion has had a significant impact on how Christianity is practised by indigenous people, particularly in more remote rural areas (Derose et al., 2010). The legacy of a 36-year civil war is weak governance and widespread violence, evidenced by high rates of organised crime and the growth of gang culture (Bjune, 2016; Escobar-Chew, 2013; UNICEF, 2016).

## Measures and Procedure

Development of the scale followed the sequence commended in Koenig and Zaben (2021), including: definition of domains, generation of items, consideration of content validity, translation and back translation to confirm consistency of meaning, pretesting of items, scale administration, item reduction, extraction of factors through exploratory factor analysis, confirmation of dimensions through confirmatory factor analysis, examination of internal consistency using Cronbach’s alpha test and preliminary confirmation of face and construct validity (future steps for examining differing forms of validity are noted in the Discussion).

A list of 32 potential survey items was drafted drawing from pre-existing surveys used to evaluate faith leader and wider faith community trainings and from literature relevant to the themes and issues that programming in this area sought to explicitly address (Eyber et al., 2018; World Vision, 2015). During the development of the measure, items were informally and thematically grouped and numbered with respect to four broad domains: (1) ‘knowledge’; (2) ‘attitudes’; (3) ‘practices’; and (4) ‘theological reflection’. ‘Knowledge’ items included questions focussed on participants’ knowledge of the relevance of birth registration, the impacts of punishments on children and the relevant reporting mechanisms and laws associated with child protection and child rights. ‘Attitude’ items included questions regarding child marriage, child labour, and the reporting of child abuse. ‘Practice’ questions addressed the practices of religious groups, faith leaders or their spouses relevant to child protection, such as reporting child abuse to authorities or meeting parents to discuss concerns. Finally, ‘theological reflection’ items explicitly probed how religious beliefs influenced approaches to childhood and child protection, linking to religious practices of prayer and understandings of scriptural teaching. All items were scored on a five-point Likert scale, with values of 1 to 5 (ranging from ‘strongly disagree’ to ‘strongly agree’). The survey was deployed across the three settings—Senegal, Uganda and Guatemala—to assess faith community orientation toward child protection issues in advance of implementation of an intervention working with faith leaders and their communities (World Vision, 2019). Across all settings participants were therefore chosen purposively (i.e. we targeted participation of faith leaders and their spouses within the communities identified) and convenience based (i.e. participation was restricted to those persons available during the data collection period). Characteristics of all participants are summarised in Table 1. Prior to administration in each setting, items were discussed with project staff with responsibility for faith community engagement and child protection work to confirm their relevance to the context. Items were then translated into relevant local languages by the local research team. The translated items were discussed with local research assistants during their training, including translation back into English, to establish appropriate contextual meanings. Subsequently, research assistants were divided into pairs and administered the revised survey items to each other in the relevant local language. Any further translation issues identified by this process were then resolved by discussion with the full research team. The survey questionnaire was then piloted in the community, with further adjustments in the use of terms until clarity and coherence of all items was established in local languages.

**Table 1:** Characteristics of survey participants by country

	Senegal^a^		Uganda		Guatemala	
	*n*	%	*n*	%	*N*	%
Total participants	161		323		216	
Christian	16	9.9	242	74.9	216^b^	100
Muslim	145	90.1	81	25.1	0	0
Males	92	57.9	191	59.1	103	47.7
Females	67	42.1	132	40.9	113	52.3
Faith leaders	92	57.9	246	76.2	141	65.3
Faith leader spouses	67	42.1	77	23.8	75	34.7

^a^Gender and role information missing for two participants

^b^Of which 160 were Catholics and 56 Evangelicals

^c^No data collected on age in Senegal and Uganda; in Guatemala, median age of faith leaders was 45 (range 26 to 70) and of spouses 42 (range 25 to 70)

In Senegal 161 surveys comprising these 32 items were administered to local faith leaders and their spouses. Participants were aware that survey administration was in the context of appraisal of child protection issues in the selected communities and the mobilisation of local faith leaders potentially to address these. Following review of survey results and participant and data collector feedback, we prepared a revised list of 40 items (including 32 of the original items, some with revised wording to reduce ambiguity, and 8 new items). This was then administered to local faith leaders and their spouses in Uganda (323 surveys), again in the context of faith leadership orientation to child protection issues in the study areas. Finally, in Guatemala 216 40-item surveys were similarly administered to faith leaders and their spouses in the selected areas.

In Senegal versions of the items were available in French, Wolof, Pulaar, and Jhahanke. In Uganda the survey items were administered in English and Luganda and in Guatemala Spanish translations were used. All surveys were administered by trained local researchers fluent in the language selected by the interviewee. Survey items were worded to reflect statements indicative of both a positive and negative child-protective disposition. For analysis purposes, negatively worded items were subsequently reverse coded such that scoring on all items related to a positive disposition toward child protection.

## Analyses

The thematic domains of ‘knowledge’, ‘attitudes’; ‘practices’ and ‘theological reflection’ were used only to elicit items providing a suitable breadth of coverage. The validity of any item groupings—and their relevance to specifying constructs relevant to measuring child-protective disposition—was ascertained through the use of factor analysis. All analyses were conducted in SPSS.

*Country-specific analyses:* With each country data set we reviewed results of the Kaiser–Meyer–Olkin test to assess the sampling adequacy for exploratory factor analyses, and results of the Bartlett test of sphericity to assess whether correlation matrices were identity matrices (Fabrigar & Wegener, 2011). We then conducted exploratory factor analyses using maximum likelihood extraction methods and varimax rotation with Kaiser normalisation. The number of factors retained was based on appraisal of scree plots for each data set and on the basis of percentage of total variance explained by the different factor solutions.

### Combined Cross-Country Analyses

We created a combined cross-country data set and applied the processes detailed above to ascertain suitability for further exploratory factor analysis.

### Comparative Country Analyses

Guided by the number of factors identified in individual country analyses and the analysis of the cross-country data set, we conducted exploratory factor analyses imposing three factor solutions on each country data set and on the combined cross-country data set. To identify items that were most consistently associated with these factors, we first conducted an iterative comparative analysis of results and removed items of limited value in explaining overall variance across each of the data sets in a stepwise manner. Two criteria were used to guide item removal. First, a stepwise deletion of ‘least fitting’ items was implemented using a 0.3 factor loading as the threshold level (i.e. all items loading onto a factor below the threshold of 0.3 in at least one country were identified for deletion). Second, given the relevance of retaining items which performed strongly (even if in only one country), we retained items with a loading of above 0.5 in at least one setting (even if they loaded below 0.3 on one or more of other countries).

The above process ensured that we removed items of least measurement relevance while retaining those items that may be contextually more relevant in specific faith settings. After this systematic process of item deletion, we conducted a confirmatory factor analysis (imposing a three-factor solution as guided by our previous analyses) and reviewed resulting model fit (using root means square error approximation (RMSEA) and the comparative fit index (CFI)). We engaged in a further systematic process of item deletion (removing items of least variance explanatory value) until model fit measures reached acceptable thresholds (RMSEA below 0.6 and CFI above 0.8).

### Ethical Approval

Queen Margaret University’s ethical review board reviewed and approved the study, with local permissions sought in each setting as per protocol.

## Results

### Exploratory Factor Analysis—Senegal

Suitability for factor analysis was confirmed by results of the Kaiser–Meyer–Olkin measure of sampling adequacy (0.690) and Bartlett’s test of sphericity χ^2^(496) = 139.132, *p* < 0.001. Initial factor analyses identified ten factors with eigenvalues above 1; however, review of the scree plot showed an inflection point in the gradient indicating an ideal factor solution of three factors, cumulatively explaining approximately 25% of variance according to extraction and rotation sums of squared loading. Goodness of fit testing of the three factor solution shows χ^2^(403) = 680.038, *p* < 0.001, suggesting testing for factor solutions with more items may be appropriate. However, neither the scree plot nor factor loading table (see Supplementary Table S1) supports this, with the modest sample size likely influencing goodness of fit scoring.

Supplementary Table S1 shows the rotated factor matrix with item loadings associated with each of these three factors. Factor 1 loads significantly on items drawn from across all four of the preliminary domains, with a common focus on attitudes toward children and their rights. Factor 2 loads on a number of items linked to explicit religious practices or religious supports for children from the ‘practice’ and ‘theological reflection’ domains. Finally, factor 3 has significant loadings on items related to activities and norms—both personal and communal—linked to the wellbeing and support of children. A total of five items—mostly linked to reporting information to authorities—did not load significantly on any of these three factors.

### Exploratory Factor Analysis—Uganda

Data were suitable for conducting exploratory factor analyses: the Kaiser–Meyer–Olkin measure of sampling adequacy being 0.759 and Bartlett’s test of sphericity χ^2^(780) = 2666.29, *p* < 0.001. Initial analyses identified thirteen factors with eigenvalues above 1; however, review of the scree plot again showed an inflection point indicating an ideal factor solution of three factors, cumulatively explaining approximately 21% of variance according to extraction and rotation sums of squared loading. As with data from Senegal, goodness of fit testing suggests models with more factors warrant exploration (χ^2^(663) = 1085.36, *p* < 0.001); however, neither the scree plot nor factor loadings (see Supplementary Table S2) supported such analyses.

Supplementary Table S2 shows the rotated factor matrix with item loadings associated with each of these three factors. Factor 1 includes many of the items loaded on factor 3 in the Senegal analysis; there is commonality in the focus on personal and communal practices and norms in dealing with children. Factor 2 resembles a similar grouping of items to factor 2 in the Senegal analysis, with a focus on community practices linked to religious belief and affiliation. Factor 3 then comprises items about attitudes to children and their rights, similar in coverage to factor 1 in the Senegal analysis. A total of six items—mostly linked to issues of discipline—did not load significantly on any of these three factors. Comparing Tables S1 and S2 it is apparent that the item pool used in each setting has factored in similar ways, suggesting emergence of constructs of relevance to both contexts.

### Exploratory Factor Analysis—Guatemala

As with Senegal and Uganda data sets, data from Guatemala was confirmed as suitable for exploratory factor analyses on the basis of the Kaiser–Meyer–Olkin measure of sampling adequacy (0.818) and Bartlett’s test of sphericity (χ^2^(780) = 2612, *p* < 0.001). Initial factor analyses identified twelve factors with eigenvalues above 1; however, the scree plot again converged on a solution of three factors, cumulatively explaining 31% of variance according to extraction and 26% by rotation sums of squared loading, respectively. As with the other country analyses, goodness of fit testing suggests exploring further factor solutions (χ^2^(663) = 1032, *p* < 0.001), but scree plots and factor loadings (see Supplementary Table S3) confirmed the appropriateness of a three-factor solution.

Supplementary Table S3 shows the rotated factor matrix with item loadings associated with each of these three factors. There are clear parallels with the factor structure of items from the other settings, although also some differences in their configuration. Factor 1, for instance, is loaded on by many items associated with Factor 1 in Senegal and Factor 3 in Uganda, although a number of items addressing protective actions and practices—such as reporting concerns to relevant authorities—load on this factor in addition to items focussed on attitudes to children and their rights. Factor 2, as in both Senegal and Uganda, again reflects a number of items related to child protection practices and their linkage to religious belief or institutions. In the context of Guatemala, Factor 3 is predominantly associated with the issues of punishment and discipline. In this instance, a total of eight items did not load significantly on any of these factors. These items were drawn from across the preliminary knowledge, attitudes and practice domains and had loaded across a number of different factors in the analyses for Senegal and Uganda.

### Exploratory Factor Analysis—Pooled Data Set

The pooled cross-country data set met the criteria for factor analysis with the Kaiser–Meyer–Olkin measure of sampling adequacy of 0.814 and a Bartlett’s test of sphericity of χ^2^(780) = 4273, *p* < 0.001. Initial factor analysis identified eleven factors with eigenvalues above 1; however, interpretation of the scree plot was consistent with a three factor solution, cumulatively explaining 28% of variance according to extraction and 22% of rotation sums of squared loading, respectively. Goodness of fit testing performed similarly to individual country solutions (χ^2^(663) = 1304, *p* < 0.001).

Supplementary Table S4 shows the rotated factor matrix with item loadings associated with each of these three factors. Pooling data across the three countries clearly not only increases the sample size for the analysis, but also substantially increases the cultural and contextual variance shaping item scoring. Drawing on interpretation of factor structures in each country setting, it is apparent that Factor 1 links items concerned with child-protective actions and practices of faith leaders and religious institutions, Factor 2 links items focussed on attitudes to children and their rights, and Factor 3 is focussed upon forms of punishment and responsibilities for discipline.

### Comparative Country Analyses and Final Measure

As noted previously, we engaged in a systematic comparison of factor structures across the individual country data sets, systematically removing items of ‘least fit’ across countries while still retaining items loading above 0.5 in individual settings. After applying these rules, the item removal process concluded at step 14, resulting in the removal of 13 items; i.e. 27 measurement items remained out of the initial 40.

We further reviewed remaining items, both their phrasings and loadings, against the factors they were grouped under and sought to retain those that best fit with the core construct suggested by each factor. Items 3g and 3f clearly did not load well on the emergent factor structure so were deleted. Items 4f, 3i and 3e approached—but were below—our loading threshold of 0.5 so were also deleted from further analyses (although we noted these items as of potential value in probing religiously informed child protection practices in the context of programming).

Initial confirmatory factor analyses on the retained set of items suggested moderately good fit (with RMSEA of 0.065 and CFI of 0.710). We therefore reviewed results and deleted items with poorest fit and least qualitative relevance when grouped under factors (items 3h, 2d, 3j, 1c and 3b, although items 3j and 3b were again noted as potential value as supplementary probes of concrete practices faith communities relevant to designing context-specific programming). Items 2d, 1c and 3h were suggestive of similar concepts as identified factors so were deleted. Post deletion of these items we re-ran the confirmatory analyses on the items loading well onto the three factors: RMSEA and CFI improved, to 0.055 and 0.834, respectively, and indicated relatively good model fit.

The measure as suggested by the above analyses is presented in Table 2. The main psychometric measure is organised around seventeen survey items grouped under three constructs which reflect wider community orientations and practices toward child care and protection, child rights, and physical punishment. The additional five items listed refer specifically to practices that the faith community may engage in to enhance child protection and child wellbeing, retained on the basis of their potential utility as probes to inform programming.

**Table 2:** Faith Community Child Protection Scale

Component	Construct	Code	Measurement item	Original item code
Psychometric measure	Construct 1: Faith community child care and protection practices	CP1	I regularly pray for children in our community that are facing difficult circumstances	4g
		CP2	I put time aside to listen carefully to the concerns of my own child/children	3c
		CP3	It is important to listen and to talk to children about their opinions	2i
		CP4	It is my religious duty to protect and support children with disability because all children are created equally by God	4a
		CP5	Reporting child abuse to a child protection committee is a good thing	2q
		CP6	I know how to report child abuse to the authorities	1f
		CP7	All children—no matter what their circumstances or behaviour—are equally precious and created in God’s image	4c
		CP8	It is important to register the birth of a child who has a disability	2l
	Construct 2: Faith community attitude toward child rights	AR1	There are laws in place that protect children	1g
		AR2	It does no harm to withhold food from a child who has disobeyed	1i
		AR3	Child rights are not acceptable, since they force us to allow practices which go against our scriptural beliefs	4i
		AR4	Long and hard hours of work in the fields doesn’t harm a child	1h
		AR5	It is more useful for boys to complete school than girls	2n
		AR6	If it was discovered that a faith leader abused a child, then they should not be exposed or penalised because they are doing God’s work	4d
	Construct 3: Faith community views around physical punishment	PP1	You sometimes need to strike a child that is misbehaving	2p
		PP2	It is my understanding that our Scriptures allow us to spank our children to discipline them	4h
		PP3	In order to bring up a child properly a child needs to be physically punished	1a
Additional items*		FP1	At Friday prayer/church services, religious leaders regularly discuss issues of children’s welfare	4f
		FP2	Our church/mosque has conducted child protection training for our staff/volunteers working with children	3i
		FP3	Members of our church/mosque meet with people from other churches/mosques to consider ways to protect the most vulnerable children in our community	3e
		FP4	Faith leaders regularly report child protection issues to the authorities	3j
		FP5	I often meet with parents of children to talk about the importance of registering a child’s birth	3b

*Recommended as focus for qualitative open questioning rather than as Likert-type closed questions

Mean full-scale and sub-scale scores for each country setting are shown in Table 3. This indicates scores to be positively skewed, but not displaying an appreciable ceiling effect, with full-scale mean scores generally around two standard deviations below the maximum score of 85 (and sub-scale scores between one and two standard deviations below the maximum). Internal consistency of full-scale scores in each setting is provided in Table 4. In Guatemala and Uganda, the settings where the full-set of 17 items was used, alphas fall in the range (0.60–0.80) suggesting moderate evidence of a meaningful latent construct of child-protective disposition underlying attained scores (Hair et al., 2006). In Senegal, the alpha is well below this level although the noted absence of four scale items is a likely contributor to this. Cronbach’s alpha is an unreliable measure of internal consistency with a small number of items; sub-scale alphas reported in Table 4 are thus provided for information only. The exploratory factor analyses reported are a more appropriate indicator of the coherence of these sub-scales (Hair et al., 2006).

**Table 3:** FCPPS Full scale and sub-scale scores by country

	Senegal*	Uganda	Guatemala
Full scale	71.29	70.50	71.90
	(sd = 6.24)	(sd = 5.84)	(sd = 7.30)
	(*N* = 154)	(*N* = 316)	(*N* = 216)
CP	36.10	36.76	35.33
	(sd = 3.47)	(sd = 2.92)	(sd = 3.28)
	(*N* = 156)	(*N* = 316)	(*N* = 216)
AR	23.77	25.94	25.55
	(sd = 3.38)	(sd = 3.30)	(sd = 3.80)
	(*N* = 159)	(*N* = 321)	(*N* = 216)
PP	10.69	7.83	11.01
	(sd = 3.15)	(sd = 2.60)	(sd = 2.84)
	(*N* = 161)	(*N* = 323)	(*N* = 216)

*Pro-rated for four missing items (one on CP, two on ACR and one on PP)

**Table 4:** Internal consistency of FCPPS full and sub-scale scores by country (Cronbach’s alpha)

	Senegal	Uganda	Guatemala
Full scale	0.502	0.653	0.772
	(N of items = 13)	(N of items = 17)	(N of items = 17)
	(*N* = 154)	(*N* = 316)	(*N* = 216)
CP	0.682	0.722	0.694
	(n items = 7)	(n items = 8)	(n items = 8)
	(*N* = 156)	(*N* = 316)	(*N* = 216)
AR	0.122	0.583	0.747
	(n items = 4)	(n items = 6)	(n items = 6)
	(*N* = 159)	(*N* = 321)	(*N* = 216)
PP	0.292	0.532	0.594
	(n items = 2)	(n items = 3)	(n items = 3)
	(*N* = 161)	(*N* = 323)	(*N* = 216)

## Discussion

Factors influencing the care and protection of children are clearly deeply culturally embedded. The use of qualitative methods that can appraise local constructions of childhood, parental and community responsibilities toward children, prevailing risks, and the role of faith leaders and others with leadership roles within communities is therefore an essential component of analysis of the protective environment for children in any setting (Rutledge & Eyber, 2019).

However, as in related areas of study, such as the deployment of religious coping strategies (Al-Hadethe et al., 2016), there are major potential benefits of developing robust quantitative measures complementing this form of contextual analysis (Koenig & Al Zaben, 2021). Specifically, there is significant value in the identification of factors consistently underlying faith communities’ approaches to child protection. First, quantitative measurement of factors shared across settings provides a potential means of comparison across time or across settings in a way that can assess the effectiveness of interventions. Second, the identification of factors that shape the disposition of faith communities toward children promises to support our conceptual understanding of mechanisms of community-based child protection.

In consequence, the identification of 17 items that proved of relevance in understanding the child-protective disposition of faith communities across three very different cultural and religious settings is of significant promise. These items suggest that child care and protection practices, attitude toward child rights and views on the use of physical punishment are consistent factors shaping the overall approach of faith communities to children’s wellbeing. The documentation of a similar factor structure relating to these issues across three settings of such geographical, cultural and religious divergence is particularly noteworthy. The integration of items explicitly referring to religious or spiritual concerns—drawn from the initial ‘theological reflection’ set of items—into each of the factors of the measure is also an important finding. Reflecting on the implications of religious teaching or spiritual practice does not lie outside of and distinct from knowledge, attitudes and practices regarding child protection but is firmly embedded within such considerations. The recognition of faith being inseparable from understanding within communities with strong religious affiliation and belief has important implications for programmatic interventions seeking to shape behaviour and social norms (LWF & IRW, 2018).

As noted, the consistency in factor structures identified through exploratory factor analyses and the moderate Cronbach’s alphas secured for the full-scale are encouraging regarding the internal reliability of the measure (i.e. the extent to which the measure is consistent within itself). The extent to which the measure is consistent from one use to another—that is, its external reliability—was not addressed in the current study, however, and is an appropriate focus for future research. In terms of evidence regarding validity—that is the measure identifying substantive issues of relevance regarding child protection views and practices—we note that significant differences in FCCPS scores could be triangulated with qualitative findings across the three country settings (Jailobaeva et al., 2021). Eyber et al. (2021) found a preliminary version of the FCCPS to detect change in child protection knowledge, attitudes and practices in line with child protection programme goals with faith communities. Further work is clearly required, however, to establish the wider validity of the FCCPS as a child protection measure with faith communities.

### Limitations and Robustness

We acknowledge our survey data was of a five point Likert scale and therefore does not fully meet the recommended criteria for the use of exploratory factor analyses using maximum likelihood extraction methods. However, the latter methods have been shown to be relatively robust to violations (Curran et al. 1996, in Fabrigar & Wegener, 2011), and our further analyses using principal component extraction produced quasi-identical results (data not shown). The smaller sample size for the survey in Senegal—and the use of a smaller initial item set there—imposed some constraints on analysis and interpretation. Further exploration of the test–retest reliability and incremental and criterion validity of the measure is warranted (Koenig & Zaben, 2021) to inform use of the measure. Finally, faith leaders and their spouses represent a particular constituency within faith communities that may be seen to be particularly sensitised to matters of faith and religion (and, potentially, wider civic engagement) compared with faith communities as a whole. Measurement of child-protective disposition more widely across faith communities—and analysis of any differences from the views of faith community leaders—is potentially an important focus for further enquiry.

## Conclusion

The FCCPS measure provides a means to evaluate the impact of interventions targeting the strengthening of faith-community-based child protection, which has been signalled as an important strategic concern. Eyber and Palm (2019) note, for example, that evaluations of faith communities’ approaches to countering violence against children often ‘fly under the evidence radar’, remaining at an informal, anecdotal level. It is vital that intervention outcomes be documented rigorously in order to establish an evidence-base that guides decision-making and assesses social norm change as well as the sustainability of any changes that occur. At present the measure’s claims for validity rest on the face validity of its items (i.e. they are transparently related to the constructs suggested) and its construct validity (with the close replication of the factor structure across diverse settings). In due course we hope to be in a position to report on its criterion validity by documenting change in faith communities that have engaged in interventions seeking to strengthen child-protective influences (World Vision, 2019). Use of the measure in these contexts will also provide a basis for gauging levels of full-scale internal consistency and other measures of reliability.

### Implications for Practice

The analysis under-pinning the development of the measure has important programming implications. The factor structure of the FCCPS suggests three key foci for efforts to shape the child-protective influence of faith communities: attention to the needs and concerns of, and risks faced by, children; notions of child agency and rights; and appropriate strategies of discipline. This tripartite conceptualisation—complemented by a number of suggested probes to explore community practices—provides an empirically founded structure to address attitudes and practices amongst faith communities regarding children and childhood. This insight is shaping ongoing work with development and evaluation of the Channels of Hope Child Protection intervention across the three settings considered in this paper (World Vision, 2019). However, with increasing recognition of the lack of capacity within state provision in many low- and middle-income contexts to adequately protect children from abuse, neglect and exploitation, it has wider implications for the increasing number of initiatives seeking to effectively mobilise faith communities for the protection of children (Robinson & Hanmer, 2014; UNICEF, 2019).

## Supplementary Information

Below is the link to the electronic supplementary material.

Supplementary file1 (DOCX 38 KB)

## Data Availability

Supplementary files documenting analysis with respect to survey rewording, stepwise item deletion and item removal during comparative country analyses are available from the corresponding author on request.
