# Supplementary material for: Development of the Faith Community Child Protection Scale with Faith Leaders and their Spouses in Senegal, Uganda and Guatemala
Source: J Relig Health. 2022 Oct 15;62(3):2196–212. doi: 10.1007/s10943-022-01660-z (PMC10133037; doi:10.1007/s10943-022-01660-z)
Supplement: Supplementary file 1 — Supplementary file1 (DOCX 38 KB) [file 10943_2022_1660_MOESM1_ESM.docx]

**Supplementary Table S1**

*Rotated Factor Matrix for Senegal**

| Survey item | Factor loading | | |
| --- | --- | --- | --- |
|  | 1 | 2 | 3 |
| 2d. It is acceptable here for boys and girls of poor families to work in order to earn income for the family rather than attend school | .563 |  |  |
| 4h. It is my understanding that our Scriptures allow us to spank our children to discipline them | .557 |  |  |
| 4i. Child rights are not acceptable, since they force us to allow practices which go against our scriptural beliefs | .489 |  |  |
| 1g. There are laws in place that protect children | .484 |  |  |
| 4b. We do not read in scriptures about “child rights.” It is a worldly/secular concept with no foundation in scripture. | .472 |  |  |
| 4c. All children – no matter what their circumstances or behaviour - are equally precious and created in God’s image | .465 |  |  |
| 1c. Children deserve the same level of respect and support as adults | .409 |  |  |
| 1b. It is no business of anyone outside the home how a child is disciplined. | .380 |  |  |
| 2j. My children can go through the same hardships that I did because it makes them stronger. | .355 |  |  |
| 1h. Long and hard hours of work in the fields doesn’t harm a child | .320 |  |  |
| 1e. There is no value to registering the birth of a child with the authorities |  |  |  |
| 1a. In order to bring up a child properly a child needs to be physically punished |  |  |  |
| 2k. It is more important to keep good social relations than to report child abuse. |  |  |  |
| 3g. Our church/mosque does not provide information to parents and caregivers on how to ensure their children are protected against violence and abuse |  | .722 |  |
| 3f. Our church/mosque does not have someone children can speak to to obtain guidance and support if and when they need it. |  | .599 |  |
| 3e. Members of our church/mosque meet with people from other churches/ mosques to consider ways to protect the most vulnerable children in our community | .326 | .556 |  |
| 3i. Our church/mosque has conducted child protection training for our staff/volunteers working with children |  | .518 |  |
| 4a. It is my religious duty to protect and support children with disability because all children are created equally by God. |  | .432 |  |
| 4g. I regularly pray for children in our community that are facing difficult circumstances |  | .407 | .319 |
| 3b. I often meet with parents of children to talk about the importance of registering a child’s birth. |  | .346 |  |
| 2e. If I am aware that a child in the community was being physically or sexually abused, I would report it. |  | .338 |  |
| 3c. I put time aside to listen carefully to the concerns of my own child/children |  |  | .637 |
| 2q. Reporting child abuse to a child protection committee is a good thing |  |  | .524 |
| 1f. I know how to report child abuse to the authorities. |  | .322 | .454 |
| 1d. Verbally insulting a child can be just as harmful to a child as beating a child. |  |  | .445 |
| 2h. All traditional customs benefit children |  |  | -.354 |
| 2c. It is acceptable here for girls to marry before the age of 18 years. |  |  | .348 |
| 4d. If it was discovered that a faith leader abused a child, then they should not be exposed or penalized because they are doing God’s work. |  |  | -.330 |
| 3a. I seldom meet with parents to help them deal with a difficult situation related to their children. |  |  | .326 |
| 2i. It is important to listen and to talk to children about their opinions |  |  | .315 |
| 2f. It is only parent’s and close relative’s responsibility to make sure their children are protected |  |  |  |
| 2b. I would rather marry my daughter off young than see her fall pregnant before marriage. |  |  |  |

* Obtained using Maximum Likelihood extraction, varimax rotation with Kaiser normalization; items with loading below 0.3 suppressed.

**Supplementary Table S2**

*Rotated Factor Matrix for Uganda**

| Survey item | Factor loading | | | |
| --- | --- | --- | --- | --- |
|  | 1 | 2 | 3 |  |
| 2i. It is important to listen and to talk to children about their opinions | .546 |  |  |  |
| 4a. It is my religious duty to protect and support children with disability because all children are created equally by God. | .501 |  |  |  |
| 1f. I know how to report child abuse to the authorities. | .464 |  |  |  |
| 2q. Reporting child abuse to a child protection committee is a good thing | .460 |  |  |  |
| 2e. If I am aware that a child in the community was being physically or sexually abused, I would report it. | .427 |  |  |  |
| 4c. All children – no matter what their circumstances or behaviour - are equally precious and created in God’s image | .419 |  |  |  |
| 3c. I put time aside to listen carefully to the concerns of my own child/children | .407 | .368 |  |  |
| 1g. There are laws in place that protect children | .376 | .343 |  |  |
| 1e. There is no value to registering the birth of a child with the authorities | .354 |  |  |  |
| 2c. It is acceptable here for girls to marry before the age of 18 years. | .337 |  |  |  |
| 1b. It is no business of anyone outside the home how a child is disciplined. | .332 |  | .302 |  |
| 2l. It is important to register the birth of a child who has a disability. | .328 |  |  |  |
| 3f. Our church/mosque does not have someone children can speak to to obtain guidance and support if and when they need it. | .327 |  |  |  |
| 2d. It is acceptable here for boys and girls of poor families to work in order to earn income for the family rather than attend school | .317 |  |  |  |
| 2p. You sometimes need to strike a child that is misbehaving. |  |  |  |  |
| 3b. I often meet with parents of children to talk about the importance of registering a child’s birth. |  | .662 |  |  |
| 4f. At Friday prayer/church services, religious leaders regularly discuss issues of children’s welfare. |  | .519 |  |  |
| 3i. Our church/mosque has conducted child protection training for our staff/volunteers working with children |  | .518 |  |  |
| 3e. Members of our church/mosque meet with people from other churches/ mosques to consider ways to protect the most vulnerable children in our community |  | .516 |  |  |
| 3h. We have birth certificates for all the children in our household. |  | .446 |  |  |
| 3j. Faith leaders regularly report child protection issues to the authorities. |  | .428 |  |  |
| 4g. I regularly pray for children in our community that are facing difficult circumstances | .366 | .414 |  |  |
| 3g. Our church/mosque does not provide information to parents and caregivers on how to ensure their children are protected against violence and abuse |  | .363 |  |  |
| 1j. Adults should let children participate in decisions that affect their lives. |  |  |  |  |
| 1h. Long and hard hours of work in the fields doesn’t harm a child |  |  | .509 |  |
| 2f. It is only parent’s and close relative’s responsibility to make sure their children are protected |  |  | .435 |  |
| 2n. It is more useful for boys to complete school than girls. |  |  | .425 |  |
| 2b. I would rather marry my daughter off young than see her fall pregnant before marriage. |  |  | .419 |  |
| 4d. If it was discovered that a faith leader abused a child, then they should not be exposed or penalized because they are doing God’s work. |  |  | .417 |  |
| 2j. My children can go through the same hardships that I did because it makes them stronger. |  |  | .406 |  |
| 4i. Child rights are not acceptable, since they force us to allow practices which go against our scriptural beliefs |  |  | .370 |  |
| 1a. In order to bring up a child properly a child needs to be physically punished |  |  | .369 |  |
| 4b. We do not read in scriptures about “child rights.” It is a worldly/secular concept with no foundation in scripture. |  |  | .368 |  |
| 1i. It does no harm to withhold food from a child who has disobeyed. |  |  | .340 |  |
| 2h. All traditional customs benefit children |  |  | .333 |  |
| 1c. Children deserve the same level of respect and support as adults |  |  | .315 |  |
| 2k. It is more important to keep good social relations than to report child abuse. |  |  |  |  |
| 4h. It is my understanding that our Scriptures allow us to spank our children to discipline them |  |  |  |  |
| 3a. I seldom meet with parents to help them deal with a difficult situation related to their children. |  |  |  |  |
| 1d. Verbally insulting a child can be just as harmful to a child as beating a child. |  |  |  |  |

* Obtained using Maximum Likelihood extraction, varimax rotation with Kaiser normalization; items with loading below 0.3 suppressed.

**Supplementary Table S3**

*Rotated Factor Matrix for Guatemala**

| Survey items | Factor loadings | | |
| --- | --- | --- | --- |
|  | 1 | 2 | 3 |
| 1g. There are laws in place that protect children | .677 |  |  |
| 3c. I put time aside to listen carefully to the concerns of my own child/children | .649 |  |  |
| 4c. All children – no matter what their circumstances or behaviour - are equally precious and created in God’s image | .647 |  |  |
| 4g. I regularly pray for children in our community that are facing difficult circumstances | .617 |  |  |
| 2i. It is important to listen and to talk to children about their opinions | .600 |  |  |
| 4a. It is my religious duty to protect and support children with disability because all children are created equally by God. | .592 |  |  |
| 3h. We have birth certificates for all the children in our household. | .576 |  |  |
| 1c. Children deserve the same level of respect and support as adults | .530 |  |  |
| 2l. It is important to register the birth of a child who has a disability. | .529 |  |  |
| 3j. Faith leaders regularly report child protection issues to the authorities. | .508 |  | .321 |
| 2q. Reporting child abuse to a child protection committee is a good thing | .494 |  |  |
| 2e. If I am aware that a child in the community was being physically or sexually abused, I would report it. | .422 |  |  |
| 4f. At Friday prayer/church services, religious leaders regularly discuss issues of children’s welfare. | .361 |  |  |
| 2h. All traditional customs benefit children |  |  |  |
| 2j. My children can go through the same hardships that I did because it makes them stronger. |  |  |  |
| 3b. I often meet with parents of children to talk about the importance of registering a child’s birth. |  |  |  |
| 3g. Our church/mosque does not provide information to parents and caregivers on how to ensure their children are protected against violence and abuse |  | .564 |  |
| 4d. If it was discovered that a faith leader abused a child, then they should not be exposed or penalized because they are doing God’s work. |  | .557 |  |
| 3f. Our church/mosque does not have someone children can speak to to obtain guidance and support if and when they need it. |  | .548 |  |
| 2n. It is more useful for boys to complete school than girls. | .380 | .530 |  |
| 4i. Child rights are not acceptable, since they force us to allow practices which go against our scriptural beliefs |  | .508 |  |
| 1d. Verbally insulting a child can be just as harmful to a child as beating a child. | .389 | .455 |  |
| 1h. Long and hard hours of work in the fields doesn’t harm a child |  | .454 |  |
| 1i. It does no harm to withhold food from a child who has disobeyed. | .433 | .450 |  |
| 1e. There is no value to registering the birth of a child with the authorities |  | .417 |  |
| 4b. We do not read in scriptures about “child rights.” It is a worldly/secular concept with no foundation in scripture. |  | .413 |  |
| 2k. It is more important to keep good social relations than to report child abuse. |  | .402 |  |
| 2f. It is only parent’s and close relative’s responsibility to make sure their children are protected |  | .354 |  |
| 1b. It is no business of anyone outside the home how a child is disciplined. |  | .307 |  |
| 3a. I seldom meet with parents to help them deal with a difficult situation related to their children. |  |  |  |
| 1f. I know how to report child abuse to the authorities. |  |  |  |
| 1j. Adults should let children participate in decisions that affect their lives. |  |  |  |
| 2p. You sometimes need to strike a child that is misbehaving. |  |  | .594 |
| 4h. It is my understanding that our Scriptures allow us to spank our children to discipline them |  |  | .566 |
| 1a. In order to bring up a child properly a child needs to be physically punished |  |  | .439 |
| 3i. Our church/mosque has conducted child protection training for our staff/volunteers working with children |  |  | .409 |
| 2b. I would rather marry my daughter off young than see her fall pregnant before marriage. |  |  | .331 |
| 2d. It is acceptable here for boys and girls of poor families to work in order to earn income for the family rather than attend school |  |  |  |
| 2c. It is acceptable here for girls to marry before the age of 18 years. |  |  |  |
| 3e. Members of our church/mosque meet with people from other churches/ mosques to consider ways to protect the most vulnerable children in our community |  |  |  |

* Obtained using Maximum Likelihood extraction, varimax rotation with Kaiser normalization; items with loading below 0.3 suppressed.

**Supplementary Table S4**

*Rotated Factor Matrix with Pooled Dataset***^*^**

| Survey items | Factor loadings | | |
| --- | --- | --- | --- |
|  | 1 | 2 | 3 |
| 3c. I put time aside to listen carefully to the concerns of my own child/children | .558 |  |  |
| 4g. I regularly pray for children in our community that are facing difficult circumstances | .533 |  |  |
| 2i. It is important to listen and to talk to children about their opinions | .515 |  |  |
| 3j. Faith leaders regularly report child protection issues to the authorities. | .509 |  |  |
| 4f. At Friday prayer/church services, religious leaders regularly discuss issues of children’s welfare. | .491 |  |  |
| 1g. There are laws in place that protect children | .459 |  |  |
| 4a. It is my religious duty to protect and support children with disability because all children are created equally by God. | .459 |  |  |
| 3b. I often meet with parents of children to talk about the importance of registering a child’s birth. | .443 |  |  |
| 2q. Reporting child abuse to a child protection committee is a good thing | .441 |  |  |
| 2e. If I am aware that a child in the community was being physically or sexually abused, I would report it. | .423 |  |  |
| 3i. Our church/mosque has conducted child protection training for our staff/volunteers working with children | .402 |  | -.329 |
| 2l. It is important to register the birth of a child who has a disability. | .387 |  |  |
| 3e. Members of our church/mosque meet with people from other churches/ mosques to consider ways to protect the most vulnerable children in our community | .372 |  |  |
| 4c. All children – no matter what their circumstances or behaviour - are equally precious and created in God’s image | .370 |  |  |
| 1f. I know how to report child abuse to the authorities. | .362 |  |  |
| 3g. Our church/mosque does not provide information to parents and caregivers on how to ensure their children are protected against violence and abuse |  |  |  |
| 3a. I seldom meet with parents to help them deal with a difficult situation related to their children. |  |  |  |
| 2n. It is more useful for boys to complete school than girls. |  | .450 |  |
| 1h. Long and hard hours of work in the fields doesn’t harm a child |  | .448 |  |
| 4i. Child rights are not acceptable, since they force us to allow practices which go against our scriptural beliefs |  | .443 |  |
| 1i. It does no harm to withhold food from a child who has disobeyed. |  | .441 |  |
| 4b. We do not read in scriptures about “child rights.” It is a worldly/secular concept with no foundation in scripture. |  | .416 |  |
| 2k. It is more important to keep good social relations than to report child abuse. |  | .343 |  |
| 2j. My children can go through the same hardships that I did because it makes them stronger. |  | .335 |  |
| 4d. If it was discovered that a faith leader abused a child, then they should not be exposed or penalized because they are doing God’s work. |  | .325 |  |
| 2b. I would rather marry my daughter off young than see her fall pregnant before marriage. |  | .324 |  |
| 2d. It is acceptable here for boys and girls of poor families to work in order to earn income for the family rather than attend school |  | .305 |  |
| 1c. Children deserve the same level of respect and support as adults |  | .305 |  |
| 1e. There is no value to registering the birth of a child with the authorities |  | .303 |  |
| 3f. Our church/mosque does not have someone children can speak to to obtain guidance and support if and when they need it. |  | .301 |  |
| 2h. All traditional customs benefit children |  |  |  |
| 1d. Verbally insulting a child can be just as harmful to a child as beating a child. |  |  |  |
| 2p. You sometimes need to strike a child that is misbehaving. |  |  | -.714 |
| 1j. Adults should let children participate in decisions that affect their lives. |  |  | .520 |
| 4h. It is my understanding that our Scriptures allow us to spank our children to discipline them |  |  | -.474 |
| 3h. We have birth certificates for all the children in our household. | .326 |  | -.446 |
| 2f. It is only parent’s and close relative’s responsibility to make sure their children are protected |  | .334 | .440 |
| 2c. It is acceptable here for girls to marry before the age of 18 years. |  |  | .423 |
| 1b. It is no business of anyone outside the home how a child is disciplined. |  | .316 | .334 |
| 1a. In order to bring up a child properly a child needs to be physically punished |  |  | -.332 |

* Obtained using Maximum Likelihood extraction, varimax rotation with Kaiser normalization; items with loading below 0.3 suppressed
